# Mathematical Model for Oil Recovery Prediction of Polymer Microsphere Conformance Control Based on the Stream Tube Method

**DOI:** 10.3390/ma16041476

**Published:** 2023-02-09

**Authors:** Wenyue Zhao, Huo Tang, Fan Lu, Shuai Hu, Tongjing Liu, Nannan Li, Renzhi Song

**Affiliations:** 1 College of Mathematics and Computer Science, Chifeng University, 1 Yingbin Road, Hongshan District, Chifeng 024000, China; 2 The Unconventional Oil and Gas Institute, China University of Petroleum (Beijing), 18 Fuxue Road, Changping District, Beijing 102249, China; 3 Fengdikeng Working Area, No. 5 Oil Production Plant of Petrochina Changqing Oilfield Company, 1189 Jinghuan South Road, Gaoling District, Xi’an 710064, China; 4 Irap Branch, Greatwall Drilling Company (GWDC), CNPC, 101 Anli Road, Chaoyang District, Beijing 102249, China

**Keywords:** polymer microspheres’ conformance control, oil recovery prediction model, inverted nine-spot rhombus pattern, areal sweep efficiency, stream tube method

## Abstract

Oil recovery is an essential parameter for reservoir development performance evaluation, but there is no specific research on the theoretical oil recovery prediction model of polymer microspheres (PMs)’ conformance control. This research aims to establish an oil recovery prediction model that depends on the definition of oil recovery based on stream tube theory. PMs’ enhanced oil recovery mechanism is to plug the pore throat to expand the swept area. The assumption that the stream tube between injection and production wells is trapezoidal is proposed. Based on this premise, the area sweep efficiency equation suitable for the rhombus inverse nine-spot well pattern is established based on the stream tube theory. The vertical sweep efficiency equation is optimized by introducing the equivalent mobility ratio. Additionally, the model’s adaptability and validity are studied. The analysis results show that oil recovery decreases with increasing injection rate, injection concentration, and PMs size but increases with the increasing injection period. The theoretical oil recovery is 1.37%, and the actual oil recovery of the field application is 1.22%, with an error of 0.15%. This model has good consistency with the actual physical process of the field application. The oil recovery prediction model can provide oil recovery and optimize PMs’ conformance control injection scheme. This study fills the gap in the mathematical model for oil recovery prediction of PMs’ conformance control.

## 1. Introduction

Low-permeability reservoirs are an essential type of reservoir globally, and are mainly distributed throughout Mexico, China, Algeria, Egypt, the United States, and other countries [1,2]. Currently, complex and challenging low-permeability oil fields account for an increasing proportion of those in China, and a considerable number of oil fields are working at a low production rate and production efficiency [3,4,5]. Therefore, developing low-permeability oil reservoirs is significant for China’s energy supply [6,7].

In low-permeability reservoirs, severe heterogeneity and an inconsistent water flooding displacement profile will significantly affect water flooding performance [8]. In the process of water flooding, a quick water breakthrough will occur because the injected water rushes along existing micro-cracks, leading to a shorter stable production period [9]. Unlike the chain-like polymer repeatedly connected by specific structural units, polymer microspheres (PMs) are spherical polymer composite materials with diameters ranging from nanometers to micrometers, a high specific surface area, high reactivity, and other unique physical, chemical, and biological properties [10]. PMs show good conformance control ability as salt tolerance materials [11,12]. In the reservoir migration process, PMs can be plugging, deformation, migration, and second plugging, and have an excellent ability to improve formation heterogeneity and stop or slow down one-way water rush [13]. PMs can not only reduce the water cut rising of water breakthrough well; they also have double functions of conformance control and oil displacement. Increasing water injection volume can achieve a long-term stable oil production period and the ultimate goal of enhanced recovery [14,15]. PMs conformance control technology has been applied to Bohai, Shengli, Changqing, and Jidong oilfields and has achieved good performance of oil incrementation [16,17,18].

The oil recovery factor is one of the essential parameters to describe the effect of oilfield development performance. The primary means of accessing oil recovery include numerical simulation, laboratory experiments, empirical formulas, and theoretical equations [19,20,21,22,23]. However, for PMs conformance control, oil recovery efficiency cannot be predicted by numerical simulation because numerical simulation technology cannot characterize the enhanced oil recovery mechanism of plugging, deformation, migration, and second plugging. For the laboratory experiment method, due to the apparent differences between the scale of the core and the field, and the relatively simple distribution of core pore-throat, the oil recovery obtained from the laboratory is significantly different from the actual field application effect. Therefore, the mathematical model of oil recovery can only be established based on the definition of oil recovery.

The theoretical equation based on the oil recovery factor’s definition is fundamental to oil production forecasting and the development of performance evaluation. The oil recovery factor is determined by oil displacement efficiency, areal sweep efficiency, and vertical sweep efficiency [24]. Oil displacement efficiency at different water cuts can be resolved with an oil–water relative permeability curve and extreme oil displacement efficiency [25,26,27].

Areal sweep efficiency is the critical parameter to evaluate the displacement performance of area well pattern [28]. Many scholars in China and other countries have conducted corresponding research on areal sweep efficiency. Before 2000, research methods for the area sweep efficiency included laboratory experiments, field data analysis, numerical simulation, probability theory, and dimensional analysis [29,30,31,32,33,34,35,36,37]. Since 2010, the stream tube method has been used to study the relationship between area sweep efficiency and time [38,39,40,41,42,43,44,45]. The most significant advantage of streamlined simulation is its fast computing speed, high precision, and good visibility [46].

Vertical sweep efficiency is an essential parameter for evaluating the water flooding effect in the thick reservoir. The standard approach to measure vertical sweep efficiency includes a multilayer core flooding experiment and a numerical model [47,48,49,50,51]. DeSouza and Qitai Yu proposed the equation of vertical sweep efficiency calculation considering the mobility ratio, permeability variation coefficient, and the water–oil ratio [24,32]. However, for PMs flooding, the primary enhanced oil recovery mechanism is significantly different from polymer flooding, as PMs cannot increase the mobility ratio but do block the pore throat. Therefore, the vertical calculation method should be optimized to present the influence of throat blocking.

There needs to be more specific research on the theoretical oil recovery prediction model of PMs’ conformance control. This research aims to establish an oil recovery prediction model depending on the definition of oil recovery and stream tube theory. PMs’ enhanced oil recovery mechanism is to plug the pore throat to expand the swept area. The assumption that the stream tube between the injection and production wells is trapezoidal is proposed. Based on this premise, an area sweep efficiency equation suitable for the rhombus inverse nine-spot well pattern is established based on the stream tube theory. The vertical sweep efficiency equation is optimized by introducing the equivalent mobility ratio. Additionally, the model’s adaptability and validity are studied. This model not only provides a faster and more accessible tool to predict and evaluate the production performance of the PMs flooding process, but also could be used to optimize the injection scheme of PMs’ conformance control. The study of this paper fills the gap in the mathematical model for the oil recovery prediction of polymer microspheres’ conformance control.

## 2. Theoretical Model Establishment

### 2.1. Stream Tube Model

**Figure 1:**
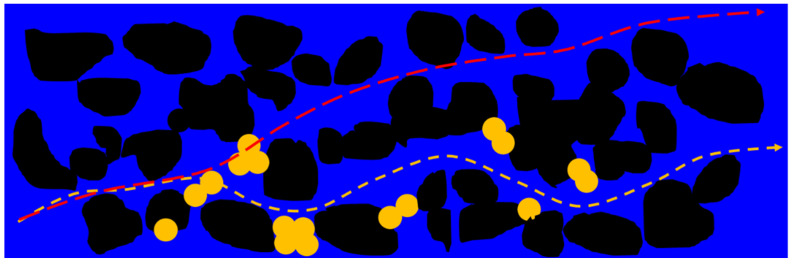
Enhanced oil recovery mechanism schematic diagram of polymer microsphere.

**Figure 2:**
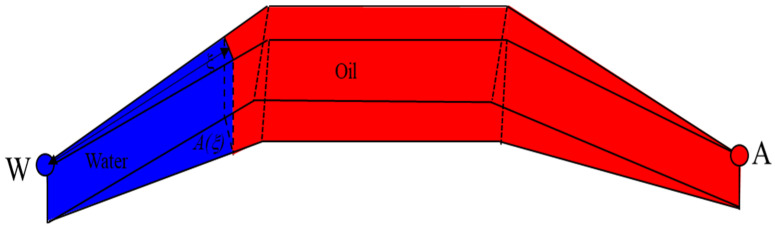
Stream tube model.

The stream tube method assumes that a non-intersecting flow pipe can express the reservoir’s physical displacement mechanism in water flooding between injection and production wells [52]. Multiple flow pipes are regularly distributed between injection and production wells, and each flow pipe is independent and parallel without interference [53,54,55]. Fluid flows in a tube without material exchange. In the conformance control process of PMs, it is assumed that there is a pipe between the injection well and the production well. The PMs block the large throats that allow the injected water to flow into the small channel within the plugging area [56,57]. After multiple bypasses of injected water, the overall flow trend tends to migrate to the production well in a straight line. Therefore, in the microsphere plugging area, the pipe is considered a straight line, and the flow path of injected water is shown in Figure 1. After flowing through the plugging area, the injected water cannot continue to cruise around and still flows to the production wells along the flow trend formed in the water flooding stage. The shape factor is used to consider the shape influence of each flow pipe. The flow pipe between injection and production wells is approximately trapezoidal, and the flow pipe between injection and production wells is shown in Figure 2.

### 2.2. Oil Displacement Efficiency

The ultimate oil recovery factor is the multiplication of sweep efficiency and displacement efficiency, and the mathematical expression is shown in Equation (1) [25]. The sweep coefficient and oil displacement efficiency are two main parameters for predicting oil recovery in water-flooding oilfields.

(1) $$E_{R}=E_{D}\cdot E_{V}$$

where *E_R_* is the oil recovery, *E_D_* is the oil displacement efficiency, and *E_V_* is the volume sweep efficiency. 

Volume sweep efficiency refers to the percentage of injected water volume in the swept area compared to the total reservoir volume. Usually, it is expressed by the product of vertical sweep efficiency *E_Z_* and areal sweep efficiency *E_A_* [24]. The expression of the sweep coefficient is:

(2) $$E_{V}=E_{A}\cdot E_{Z}$$

where *E_A_* is areal sweep efficiency, and *E_Z_* is vertical sweep efficiency.

(3) $$E_{R}=E_{D}\cdot E_{A}\cdot E_{Z}$$

The oil displacement efficiency *E_D_* is the ratio of oil produced to the petroleum geological reserves in the affected area. PMs improve oil recovery by plugging channels to expand the water sweep efficiency. The primary displacement fluid is water, so the oil displacement efficiency is consistent with the water displacement efficiency. According to the reservoir permeability curve combined with the water content formula, the water saturation of the reservoir at different water contents can be calculated, and the oil displacement efficiency can be obtained.

At present, the oil–water relative permeability curve is often expressed in exponential form, under the condition of oil and water two-phase seepage; *K_ro_*/*K_rw_* and *S_w_* are semi-logarithmic linear relationships [26].

(4) $$\frac{K_{ro}}{K_{rw}}=ae^{-bS_{w}}$$

According to the partial flow equation, without considering the influence of gravity and capillary force, the moisture content can be expressed as

(5) $$f_{w}=\frac{1}{1+\frac{\mu_{w}}{\mu_{o}}\frac{K_{ro}}{K_{rw}}}$$

So, the relationship between water content and water saturation is as follows:

(6) $$S_{w}=\frac{\ln a-\ln\left(\frac{1-f_{w}}{f_{w}}\right)-\ln\frac{\mu_{o}}{\mu_{w}}}{b}$$

In polymer microsphere flooding, we assume that the injected water enters the original unreached area, and the oil saturation in the unreached area is the initial oil saturation. The oil displacement efficiency formula of the reservoir with different water saturations is expressed as:

(7) $$E_{D}=\frac{1-S_{w}-S_{or}}{1-S_{wi}}$$

where *S_wi_* is the initial water saturation, *S_w_* is the current water saturation, and *S_or_* is the residual oil saturation.

Placing Equation (6) into Equation (7), the oil displacement efficiency at different water cuts can be calculated by the following equation:

(8) $$E_{D}=\frac{1-\frac{\ln a-\ln\left(\frac{1-f_{w}}{f_{w}}\right)-\ln\frac{\mu_{o}}{\mu_{w}}}{b}-S_{or}}{1-S_{wi}}$$

As we know, both vertical sweep efficiency and areal sweep efficiency are essential. They can affect oil recovery significantly when the injected fluid cannot improve the driving efficiency [47]. 

Among the two factors, areal sweep efficiency is more important than vertical sweep efficiency for the thin thickness of a low permeability reservoir. Therefore, the improvement potential of vertical sweep efficiency is limited, but the possibility of areal sweep efficiency is the opposite. Thus, this paper mainly focuses on the study of areal sweep efficiency.

### 2.3. Areal Sweep Efficiency

**Figure 3:**
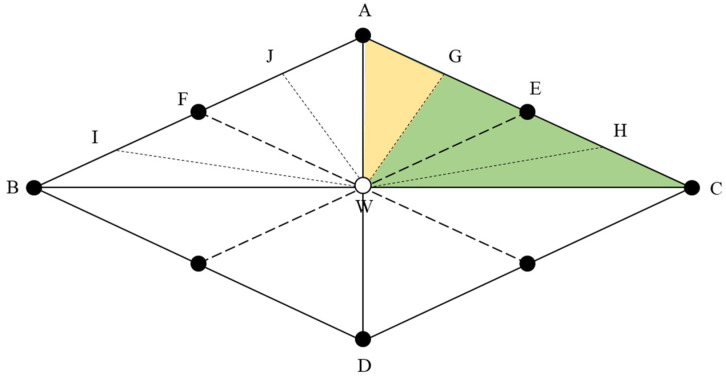
Diamond-shaped reverse nine-spot well pattern.

**Figure 4:**
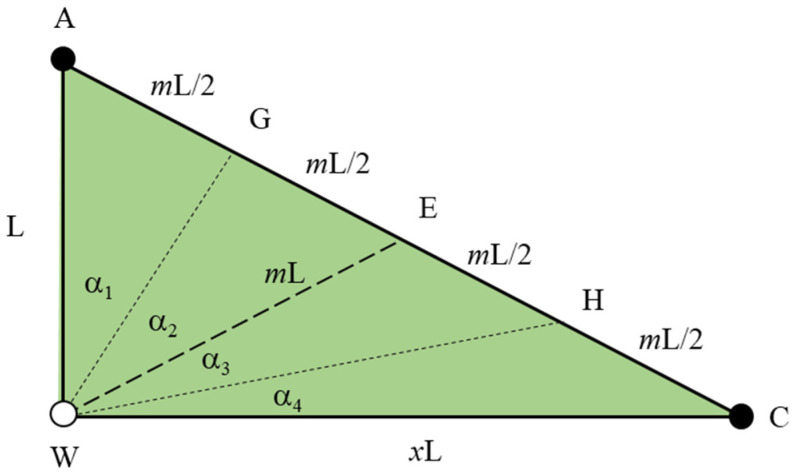
The injection–production unit of the rhombic inverted nine-point well pattern.

The model assumes that the reservoir is homogeneous. There is a flow pipe between the oil well and the water well; the flow pipe is piston water flooding oil. The compressibility of formation rocks and fluids is not considered. An inverted nine-spot pattern mainly develops low-permeability reservoirs. One well group in the inverted nine-spot pattern can be divided into four injection-production units and 16 seepage units, as shown in Figure 3. Since the rhombic inverse nine-point is a regular symmetric well pattern, the seepage unit’s area sweep coefficient can represent the well pattern’s sweep coefficient. To obtain the area sweep coefficient of a seepage unit, the seepage unit AWC is divided into four research areas, namely AWG GWE EWH and HWC. These four areas could obtain areal sweep efficiency with the same calculation method. Therefore, we take AWG as an example to deduce the areal sweep efficiency calculation model. The diamond inverted nine-spot well pattern injection–production unit is shown in Figure 4. According to the above assumptions, multiple flow pipes are established between injection wells and production wells. By analyzing the nine-point well pattern’s fluid seepage characteristics and symmetry in Figure 3, the seepage unit AWG in the nine-point well pattern is taken as the calculation unit.

**Figure 5:**
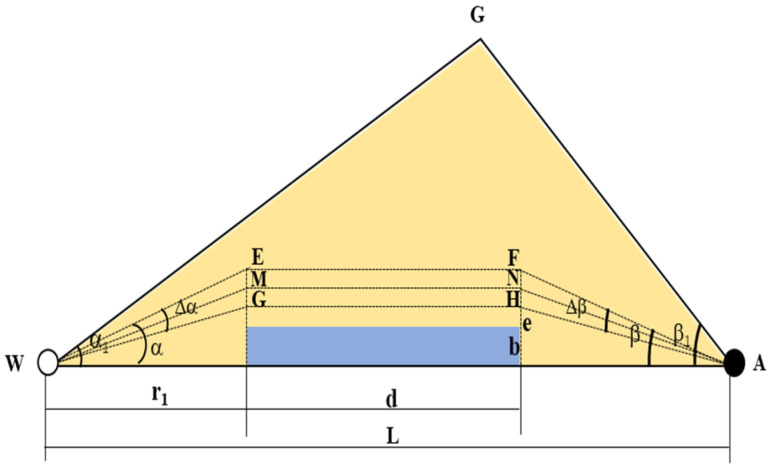
Seepage unit of rhombic inverted nine-spot well pattern, calculation unit of area sweep coefficient, schematic diagram of flow pipe in the calculation unit.

In the rhombic inverted nine-spot well pattern calculation unit AWG, the distance between the injection well and the short axis angle well is l, the GWA is α1, and the GAW is β1. The flow pipe infinitesimal injection well angle increment EWG Δα, a production well angle increment FAH Δβ, injection well angle variable EWA α, production well angle variable FAW β, a calculation unit, and the flow pipe between injection and production wells are shown in Figure 5.

In low-permeability reservoirs, the diamond-shaped reverse nine-point well pattern is used chiefly for reservoir development. According to the symmetry of injection and production units in the well pattern, the ASE can be determined by studying one in four injection and production units. When the microsphere enters and seals off the area with low permeability, it is assumed that the flow line between the injection and production wells is trapezoidal.

Areal sweep efficiency refers to the ratio of the flooded area to the well pattern control area. To calculate the areal sweep efficiency, we must obtain the stream tube’s length and cross-sectional area, the oil-water front’s position, and the flow equation, considering the starting pressure gradient is necessary.

After being injected into the reservoir, a plugging zone exists in which PMs block the large pore path. We assume that the length of the plugging zone is *d*, the width of the plugging area is *b* and the sweep width of subsequent injected water is *e*. The width of the plugging area can be calculated with Equation (9).

(9) $$b=r_{1}\sin\alpha_{w}$$

where *r*_1_ is the radius of the area in which the permeability increases due to water injection, obtained by well-test interpretation. *α_w_* is the angle of the water drive, obtained by the calculation method of the area spread coefficient in the water drive stage.

After blocking the throat with PMs, the width of the subsequent injection water ripple is:

(10) $$e=\frac{E_{RP}}{E_{RW}}b$$

where *E_RP_* is the oil recovery of laboratory polymer microsphere flooding experiment, and *E_RW_* is the oil recovery of laboratory water flooding experiment.

The formula of the polymer driving angle *α_f_* is as follows:

(11) $$\alpha_{f}=\tan(\frac{e}{r_{1}})$$

We assume that the injected polymer microspheres will block the whole pore volume. Therefore, the plugging length *d* can be calculated with the following equation:

(12) $$\frac{Q_{in}C_{p}C_{d}}{\rho_{p}}=2r_{1}\sin\alpha_{w}dh\phi (1-S_{w})$$

Currently, the Carman–Kozeny equation is commonly used to calculate the throat’s diameter(*r_R_*), shown as Equation (13).

(13) $$r_{p}=\sqrt{\frac{8K}{\phi }}$$

The ratio of polymer microsphere diameter and throat diameter is demonstrated as follows:

(14) $$\frac{r_{P}}{r_{R}}=C_{R}$$

By bringing Equations (9)–(11), (13) and (14) into Equation (12), and through unit conversion, we obtain the calculation equation of plugging length.

(15) $$d=\frac{Q_{in}C_{p}r_{P}^{2}C_{d}}{0.008Kh\rho_{p}r_{1}\sin\alpha_{w}C_{R}^{2}(1-S_{wi}-S_{or})}$$

where *Q_in_* is the injected volume of PMs, *C_p_* is the concentration of the polymer microsphere, *C_d_* is the expansion ratio of the polymer microsphere in the hydration experiment, *R_p_* is the diameter of PMs, *K* is reservoir permeability, h is the thickness of formation, *ρ_p_* is the density of polymer microsphere solution, *C_R_* is the ratio of polymer microsphere diameter and throat diameter, *S_wi_* is the irreducible brine saturation, *r_P_* is the diameter of polymer microsphere, and *S*_or_ is the residual oil saturation.

The flow pipe between injection and production wells is trapezoidal, so there are two inflection points. According to the geometric relationship, the length *L*_1_ between the injection well W and the first inflection point E is:

(16) $$L_{1}=\frac{r_{1}}{\cos\alpha }$$

The length *L*_2_ between the injection well W and the second inflection point F is:

(17) $$L_{2}=d+\frac{r_{1}}{\cos\alpha }$$

The length *L*_3_ of any pipe between the injection well and the production well A is:

(18) $$L_{3}=d+\frac{r_{1}}{\cos\alpha }+\frac{l-d-r_{1}}{\cos\beta }$$

where *l* is well spacing.

In the flow pipe *ξ*, the cross-section of the flow tube is shown in Equation (19).

(19) $$A(\xi )=\left\{ \begin{array}{l} 2h\xi \tan\frac{\Delta \alpha }{2},r_{w}<\xi <\frac{r_{1}}{\sin\alpha }\\ 2h\frac{r_{1}}{\sin\alpha }\tan\frac{\Delta \alpha }{2},\frac{r_{1}}{\sin\alpha }<\xi <d+\frac{r_{1}}{\sin\alpha }(1-\cos\alpha )\\ 2h(d+\frac{r_{1}}{\sin\alpha }(1-\cos\alpha )+\frac{l-d}{\cos\beta }-\xi )\tan\frac{\Delta \beta }{2},d+\frac{r_{1}}{\sin\alpha }(1-\cos\alpha )<\xi <d+\frac{r_{1}}{\sin\alpha }(1-\cos\alpha )+\frac{l-d}{\cos\beta }-r_{w} \end{array}\right.$$

In low-permeability reservoirs, the oil and water phase flow equation is the oil phase and water phase flow when the pressure gradient is started by non-piston water flooding.

The oil phase flow equation is shown as Equation (20).

(20) $$q_{t}=\frac{\frac{CK}{\mu }(\Delta P-\lambda L_{3})}{\int_{L}\frac{1}{A(\xi )}d\xi }$$

The pseudo tube equation before water channeling is shown as Equation (21).

(21) $$q_{t}=\frac{\frac{CK}{\mu }(\Delta P-\lambda L_{3})}{\frac{1}{2h\tan\frac{\Delta \alpha }{2}}\ln\frac{e}{r_{w}\sin\alpha }+\frac{d}{2h\frac{e}{\sin\alpha }\tan\frac{\Delta \alpha }{2}}+\frac{1}{2h\tan\frac{\Delta \beta }{2}}\ln\frac{l-d-r_{1}}{r_{w}\cos\beta }}$$

In the oil–water phase region, the distance from the oil–water front to the injection well *ξ* can be calculated with Equation (22):

(22) $$\int_{0}^{\xi }A(\xi )d\xi =\int_{o}^{t}\frac{q_{t}f_{w}^{\prime }(S_{wf})}{\phi }dt$$

where *f*′(*S_wf_*) is the changing rate of water cut at different water saturations.

When the oil–water front reaches the inflection point E, the time when the oil–water front comes is *t*_1_, the second inflection point F is *t*_2_, and the time when the oil–water front reaches the production well A is *t*_3_.

Assuming that the pipe area at inflection points E and F are equal, there is:

(23) $$\beta =\arctan\frac{r_{1}\tan\alpha }{l-r_{1}-d}$$

Combining Equations (18), (21) and (22), the Equation of time *t*_1_ of the oil–water front reaching E at different angles is obtained:

(24) $$t_{1}=\frac{\mu \phi \frac{r_{1}{}^{2}}{\cos^{2}\alpha }}{2CKf_{w}^{\prime }(S_{wf})}\frac{\ln\frac{r_{1}}{r_{w}\cos\alpha }+\frac{d\cos\alpha }{r_{1}}+\frac{1}{C_{1}}\ln\frac{l-d-r_{1}}{r_{w}\cos\beta }}{\Delta P-\lambda L_{3}}$$

The time when the oil–water front reaches F at different angles is *t*_2_:

(25) $$t_{2}=\frac{\mu \phi (\frac{r_{1}{}^{2}}{\cos^{2}\alpha }+\frac{2dr_{1}}{\cos\alpha })}{2CKf_{w}^{\prime }(S_{wf})}\frac{\ln\frac{r_{1}}{r_{w}\cos\alpha }+\frac{d\cos\alpha }{r_{1}}+\frac{1}{C_{1}}\ln\frac{\left(l-r_{1}-d\right)}{r_{w}\cos\beta }}{\Delta P-\lambda L_{3}}$$

The time of oil–water front reaching the production well at different angles *t*_3_:

(26) $$t_{3}=\frac{\mu \phi (\frac{r_{1}{}^{2}}{\cos^{2}\alpha }+\frac{2dr_{1}}{\cos\alpha }+(\frac{l-r_{1}-d}{\cos\beta }{)}^{2})}{2CKf_{w}^{\prime }(S_{wf})}\frac{\ln\frac{r_{1}}{r_{w}\cos\alpha }+\frac{d\cos\alpha }{r_{1}}+\frac{1}{C_{1}}\ln\frac{\left(l-r_{1}-d\right)}{r_{w}\cos\beta }}{\Delta P-\lambda L_{3}}$$

(27) $$\Delta P=P_{h}-P_{f}$$

P_h_ is the bottom hole pressure of the injection well, and *P_f_* is the bottom hole pressure of the production well, *r_w_* << *ξ*.

If *t* < *t*_1_, the position of oil–water front *ξ*_1_ is

(28) $$\xi_{1}{}^{2}=\frac{\frac{2CKf_{w}^{\prime }(S_{wf})}{\mu \phi }(\Delta P-\lambda L_{3})}{\ln\frac{e}{r_{w}\sin\alpha }+\frac{d\cos\alpha }{r_{1}}+\frac{\sin\alpha }{\sin\beta }\ln\frac{l-d-r_{1}}{r_{w}\cos\beta }}t$$

If *t*_1_ < *t* < *t*_2_, the position of oil–water front *ξ*_2_ is

(29) $$\xi_{2}=\frac{2CKf_{w}^{\prime }(S_{wf})\cos\alpha }{\mu \phi r_{1}}\frac{\Delta P-\lambda L_{3}}{\ln\frac{r_{1}}{r_{w}\cos\alpha }+\frac{d\cos\alpha }{r_{1}}+\frac{1}{C_{1}}\ln\frac{\left(l-r_{1}-d\right)}{r_{w}\cos\beta }}t+\frac{r_{1}}{2\cos\alpha }$$

If *t*_2_ < *t* < *t*_3_, the position of oil–water front *ξ*_3_ is

(30) $$\xi_{3}=\frac{r_{1}}{\cos\alpha }+d+\frac{l-r_{1}-d}{\cos\beta }-\sqrt{\frac{1}{C_{1}}(\frac{2CKf_{w}^{\prime }(S_{wf})}{\mu \phi }\frac{\Delta P-\lambda L_{3}}{\ln\frac{r_{1}}{r_{w}\cos\alpha }+\frac{d\cos\alpha }{r_{1}}+\ln\frac{\left(l-r_{1}-d\right)}{r_{w}\cos\beta }}t-\frac{r_{1}^{2}}{\cos^{2}\alpha }-\frac{2dr_{1}}{\cos\alpha })}$$

The area of the seepage unit is

(31) $$A_{\Delta AWD}=\frac{ml^{2}\sqrt{1-\frac{1}{4m^{2}}}}{4}$$

When *t* < *t*_1_, the water flooding swept area of a single stream tube is

(32) $$S_{i}=\frac{1}{2}\xi_{1}^{2}\Delta \alpha$$

According to Equation (24), the angle *α*_0_ for water drive in time *t* can be obtained by

(33) $$E_{A1}=\frac{\int_{0}^{\alpha_{0}}\frac{1}{2}\xi_{1}^{2}d\alpha }{A_{\Delta AWD}}$$

When *t*_1_ < *t* < *t*_2_, the angle *α_m_* can be determined by Equation (24). Therefore, the areal sweep efficiency is

(34) $$E_{A1}=\int_{0}^{\alpha_{m}}\frac{1}{2}\frac{e^{2}}{\sin^{2}\alpha }d\alpha +\int_{\alpha_{m}}^{\alpha_{f}}\frac{2e}{\cos\alpha }(d+\frac{e}{\sin\alpha }(1-\cos\alpha ))d\alpha$$

(35) $$E_{A1}=\frac{\sin(\alpha_{f}-\alpha_{m})\xi_{1}(\alpha_{m})\xi_{1}(\alpha_{f})+(\xi_{2}(\alpha_{m})-\frac{r_{1}}{\cos\alpha_{m}}+r_{1}+d)\cdot r_{1}\tan\alpha_{m}}{2A_{\Delta OAD}}$$

When *t*_2_ < *t* < *t*_3_, the angle *α*_n_ can be determined by Equation (25). Therefore, the areal sweep efficiency is

(36) $$E_{A1}=\int_{0}^{\alpha_{0}}\frac{1}{2}\frac{e^{2}}{\sin^{2}\alpha }d\alpha +\int_{0}^{\alpha_{0}}\frac{2e}{\cos\alpha }(d+\frac{e}{\sin\alpha }(1-\cos\alpha ))d\alpha +\int_{0}^{\beta_{0}}\frac{1}{2}\frac{{\left(l-d\right)}^{2}}{\cos^{2}\beta }d\beta$$

(37) $$E_{A1}=\frac{\begin{array}{l} \sin(\alpha_{f}-\alpha_{m})\xi_{1}(\alpha_{m})\xi_{1}(\alpha_{f})+r_{1}\cdot r_{1}\tan\alpha_{f}+(\xi_{2}(\alpha_{m})-\frac{r_{1}}{\cos\alpha_{m}}+\xi_{2}(\alpha_{n})-\frac{r_{1}}{\cos\alpha_{n}})\cdot r_{1}(\tan\alpha_{m}-\tan\alpha_{n})+(\xi_{2}(\alpha_{n})\\ -\frac{r_{1}}{\cos\alpha_{n}}+\xi_{3}(0))\cdot r_{1}\tan\alpha_{n} \end{array}}{2A_{\Delta OAD}}$$

When *t* > *t*_3_, the angle α_t_ can be determined by Equation (26); therefore, the areal sweep efficiency is

(38) $$E_{A1}=\int_{0}^{\alpha_{0}}\frac{1}{2}\frac{e^{2}}{\sin^{2}\alpha }d\alpha +\int_{0}^{\alpha_{0}}\frac{2e}{\cos\alpha }(d+\frac{e}{\sin\alpha }(1-\cos\alpha ))d\alpha +\int_{0}^{\beta_{0}}\frac{1}{2}\frac{{\left(l-d\right)}^{2}}{\cos^{2}\beta }d\beta$$

(39) $$E_{A1}=\frac{\begin{array}{l} \sin(\alpha_{f}-\alpha_{m})\xi_{1}(\alpha_{m})\xi_{1}(\alpha_{f})+r_{1}\cdot r_{1}\tan\alpha_{m}+(\xi_{2}(\alpha_{m})-\frac{r_{1}}{\cos\alpha_{m}}+\xi_{2}(\alpha_{n})-\frac{r_{1}}{\cos\alpha_{n}})\cdot r_{1}(\tan\alpha_{m}-\tan\alpha_{n})\\ +(l-r_{1}+d)r_{1}\tan\alpha_{n}-{\left(\xi_{3}(\frac{\alpha_{t}+\alpha_{n}}{2})-d-\frac{r_{1}}{\cos(\frac{\alpha_{t}+\alpha_{n}}{2})}\right)}^{2}(\alpha_{n}-\alpha_{t}) \end{array}}{2A_{\Delta OAD}}$$

According to the established calculation method of the area spread coefficient, the area spread coefficients *E_A_*_2_, *E_A_*_3_, and *E_A_*_4_ of ΔGWE, ΔEWH, and ΔHWC are calculated. The injection–production well spacing, injection well angle variable, production well angle variable, and injection–production unit area should be replaced in the calculation.

The area sweep efficiency of the injection–production unit is calculated by the area sweep coefficient of the four seepage units.

(40) $$E_{A}=\frac{E_{A1}\times A_{\Delta AWD}+E_{A2}\times A_{\Delta GWE}+E_{A3}\times A_{\Delta EWH}+E_{A4}\times A_{\Delta HWC}}{A_{\Delta AWC}}$$

where *A_△GWE_*, *A_△EWH_*, *A_△HWC_*, and *A_△AWC_* is triangle area.

### 2.4. Vertical Sweep Efficiency

The vertical sweep efficiency *c* is mainly affected by the oil flow ratio (*M*), the water–oil ratio (*F_WO_*), and the permeability coefficient of variation (*V_K_*) [32].

(41) $$Y=3.33c^{0.77}{\left(1-c\right)}^{-1.23}=\frac{\left(F_{WO}+0.4)\times (18.948-2.499V_{K}\right)}{(M+1.137-0.809V_{K})\times 10^{f(V_{K})}}$$

where:

(42) $$f(V_{K})=-0.6819+0.9735V_{K}+1.6453V_{K}^{2}$$

The mechanism of EOR by polymer flooding decreases the mobility difference between displaced and displacing fluids to reduce fingering effects. In contrast, PMs can plug high permeability channels and improve sweep efficiency, but they have little effect on displacement efficiency [56,57].

Due to the difference between the primary mechanism of spreading expansion and the polymer, the equivalent oil flow ratio is introduced to characterize the ability of PMs to adjust the profile. For PMs flooding, the resistance coefficient is the ratio of the water flow rate to the microsphere solution, so the resistance coefficient can characterize the flow rate ratio. The plugging ability evaluation experiment provides the flow rate ratio.

In the water flooding stage of laboratory experiment of the PMs’ conformance control,

(43) $$Q_{w}=\frac{K_{w}A\Delta P_{w}}{\mu_{w}l}$$

During the PMs injection’ period:

(44) $$Q_{p}=\frac{K_{p}A\Delta P_{p}}{\mu_{p}l}$$

The equivalent mobility ratio is

(45) $$M_{E}=\frac{\lambda_{w}}{\lambda_{p}}=\frac{\frac{k_{w}}{\mu_{w}}}{\frac{k_{p}}{\mu_{p}}}=\frac{\Delta P_{p}}{\Delta P_{w}}\times \frac{Q_{w}}{Q_{p}}$$

The calculation equation of vertical sweep efficiency is as follows:

(46) $$Y=3.33c^{0.77}{\left(1-c\right)}^{-1.23}=\frac{\left(F_{WO}+0.4)\times (18.948-2.499V_{K}\right)}{(M_{E}+1.137-0.809V_{K})\times 10^{f(V_{K})}}$$

## 3. Adapt Ability Analysis

In this theoretical model, five major parameters adjust the working scheme of polymer microsphere flooding, including injection period, injection rate, injection concentration, injection volume, and polymer microsphere size. These parameters remarkably affect the areal sweep efficiency and vertical sweep efficiency of the polymer microsphere flooding process. A series of sensitivity analyses are introduced to investigate how the parameters may affect the oil recovery of a square inverted nine-spot pattern. The parameters and set values are shown in Table 1, and other support parameters are shown in Table 2.

### 3.1. Injection Period

**Figure 6:**
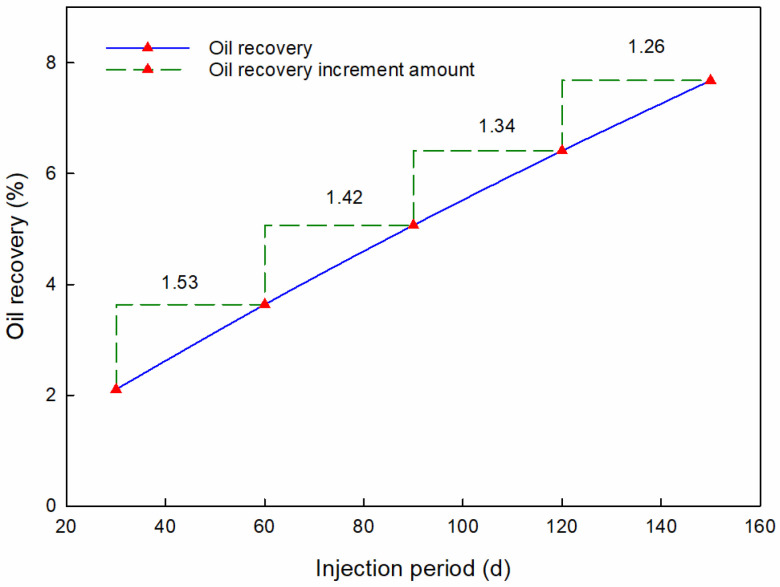
Oil recovery with different injection periods.

When the effect of the injection period is being investigated, the polymer microsphere injection rate is controlled to 40 m^3^/d, and the concentration is headed to 5000 ppm. By applying different PMs injection periods to the oil recovery calculation model, we obtained the oil recovery factor curve corresponding with the injection period, as shown in Figure 6.

According to Figure 6, the recovery factor increases with the injection period of PMs. The oil recovery rises 5.55% from 30 d of the PMs’ injection period to 150 d. It can be found that the injection period shows a significant influence on oil recovery. The longer the injection period, the greater the oil recovery. However, as oil recovery contributed, the increased PMs’ injection period declined. The oil recovery increment amount decreases from 1.53 to 1.26 as the injection period increases from 30 d to 150 d. This decrease indicates that continuing to lengthen the injection period cannot continue to improve the oil recovery.

### 3.2. Injection Rate

**Figure 7:**
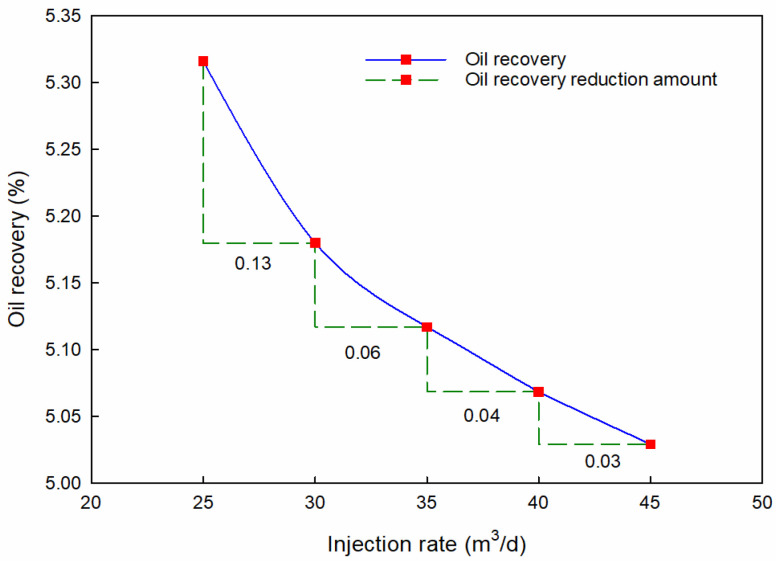
Oil recovery with different injection rates.

As for the PMs’ injection rate, the oil recovery curve for different injection rates is plotted in Figure 7. The concentration of PMs is controlled to 5000 ppm, and the total injection volume is controlled to 3.6 t.

Figure 7 shows that oil recovery decreases with the PMs’ injection rate. The oil recovery decreases from 5.31% to 5.01% as the injection rate increases from 25 m^3^/d to 45 m^3^/d. Therefore, a low injection rate is conducive to more significant recovery. Meanwhile, the oil recovery reduction amount shows the same variation trend. The oil recovery reduction amount decreased from 0.13 to 0.03. This indicates that the recovery factor can be increased significantly by lowering the injection rate when in the range of a low injection rate. When the injection rate is high, changing the injection rate to a small range has little effect on the recovery factor.

### 3.3. Injection Concentration

The concentration of the PMs’ dispersed system notably affects recovery performance in the PMs’ flooding process. The oil recovery factor–concentration relationship is studied at the same injection volume.

**Figure 8:**
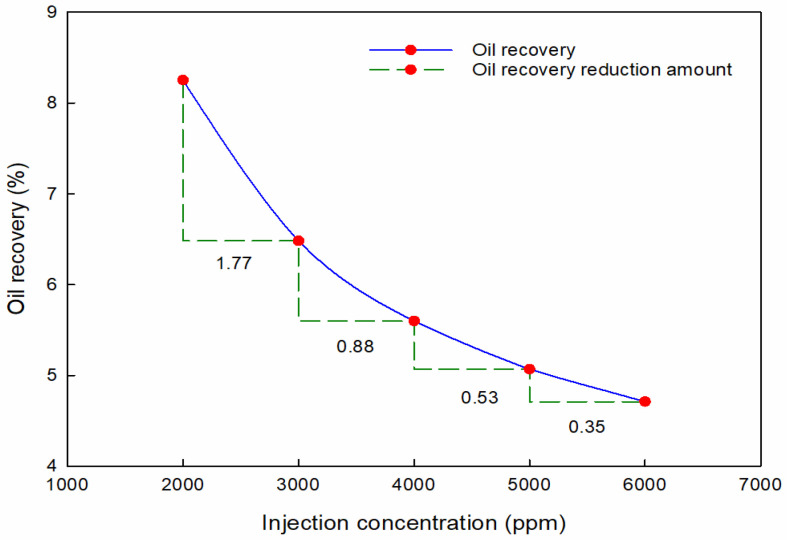
Oil recovery with different injection concentrations of PMs.

The oil recovery with different injection concentrations is shown in Figure 8. It can be seen that the oil recovery decreases with the increase in injection concentration. The final oil recovery reduces by 3.54%, with the size increasing from 2000 ppm to 6000 ppm. Therefore, low concentration is to the benefit of enhancing oil recovery. However, the best blocking rate of PMs demands the optimal injection concentration. Therefore, the injection concentration could be slightly less than the optimal injection concentration obtained from laboratory experiments to achieve the most enhanced oil recovery performance.

### 3.4. Polymer Microsphere Size

To investigate the influence of PMs’ size on conformance control performance, the injection period, injection rate, and injection concentration remained the same. In this case, the injection period is 90 d, the injection rate is 40 m^3^/d, and the injection concentration is 5000 ppm.

**Figure 9:**
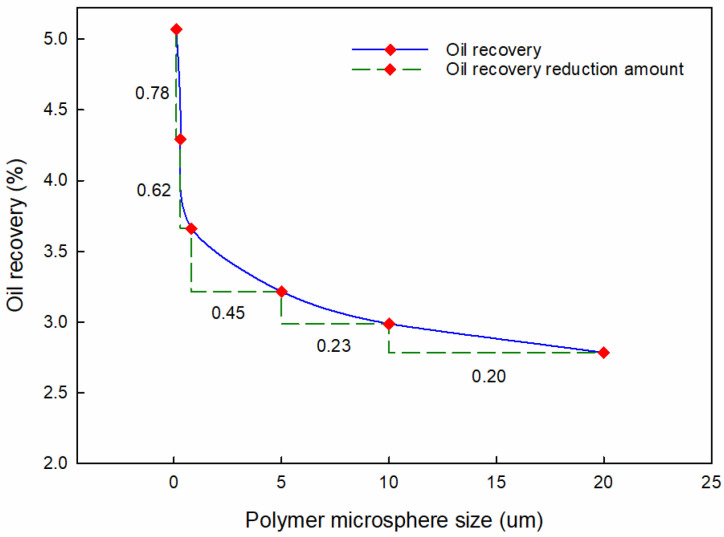
Oil recovery with different sizes of PMs.

Figure 9 shows the effect of PMs’ size on oil recovery. According to Figure 9, the oil recovery decreases with the increased PMs’ size. The oil recovery reduces by 2.315% as the size increases from 100 nm to 20 μm. The decreasing degree of oil recovery shows significant influence on the PMs’ size. The oil recovery reduces by 0.779 as the polymer microspheres’ size increases from 100 nm to 300 nm, but it only reduces by 0.254 when the PMs’ size increases from 10 μm to 20 μm. When the injected PMs have the same volume, the number of microspheres with smaller particle sizes is much larger than that with larger particle sizes. Therefore, the total volume of the tiny microspheres after hydration and expansion is more extensive. A higher plugging probability and plugging efficiency can be obtained, so smaller PMs show a much-improved oil recovery performance.

**Figure 10:**
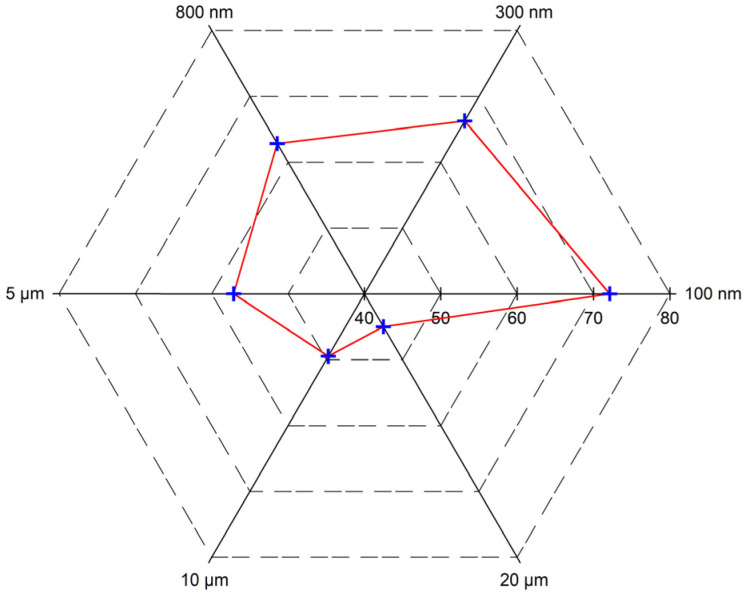
Blocking distance with different sizes of PMs.

Figure 10 shows the blocking distance of different sizes of PMs. From Figure 10, we can find that the blocking length decreases with the increase in PMs’ size. The blocking distance decreases from 72.11 m to 44.98 m as the PMs’ size increases from 100 nm to 20 μm. Tiny PMs can provide longer blocking distances. Based on the research results, it can be concluded that the smaller the size, the greater the oil recovery within the range of PMs’ size selection.

## 4. Comparison with Field Application

Oil production results estimated by the proposed theoretical model are compared with field production data collected from the PMs pilot test. The pilot test was conducted in the Changqing WYQ oil field.

### 4.1. Reservoir Background

WYQ is a typical low-permeability reservoir located in the Ordos basin. This oil field was put into development in October 1996, using an inverted nine-spot rhombus well pattern. After more than ten years of development, daily oil production decreased rapidly. PMs conformance control technology is chosen to conduct field pilot tests for its ability to improve oil recovery in low-permeability reservoirs.

**Figure 11:**
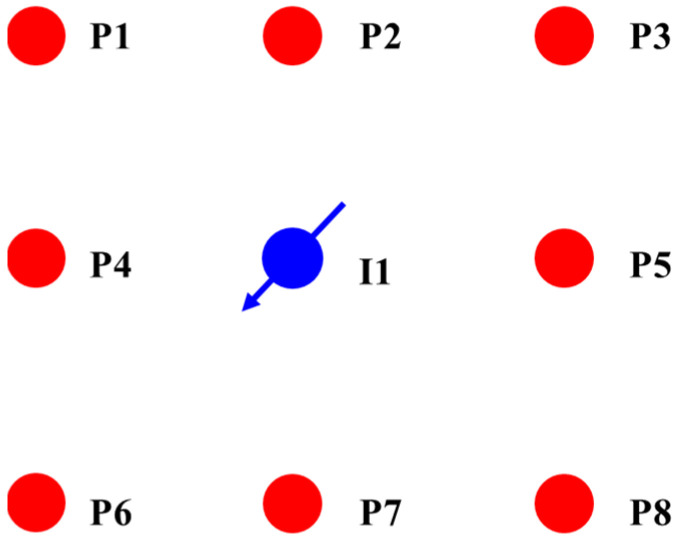
Well location of pilot test well group.

The porosity and permeability of the selected well group to conduct the pilot test are 12.64% and 28.36 mD. The reservoir temperature is 55 °C, and the formation crude oil viscosity is 1.95 MPa. The initial formation pressure of the WYQ oilfield is 13.2 MPa. Well group I1 has a water injection well and eight oil production wells; all of them are vertical wells, and the well group map is shown in Figure 11. I1 is water injection well, and P1 to P8 are oil production wells. 

**Figure 12:**
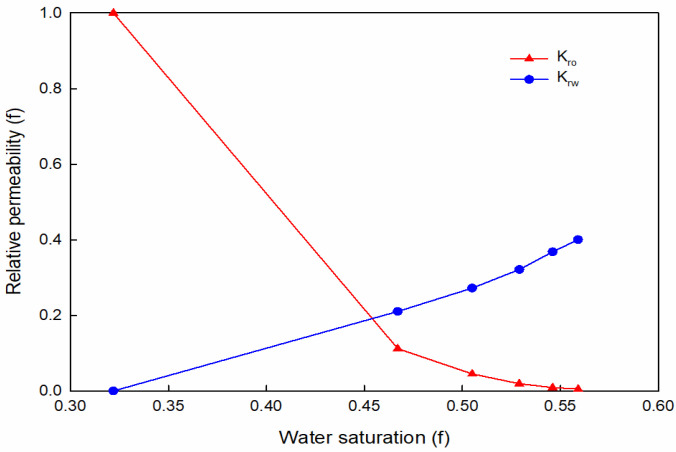
Relative permeability curve of WYQ.

The relative permeability curve is shown in Figure 12; the irreducible water saturation is 0.322, the slope of water cut at the water–oil front is 4.73, and the oil displacement efficiency is 0.42.

### 4.2. Field Application Scheme of Polymer Microsphere Flooding

The porosity and permeability of the selected well groups are 12.64% and 28.25 mD, obtained from well testing. The throat diameter calculated with the K-C equation based on the porosity and permeability is 3.36 μm. Therefore, the researchers chose PM800 as the PMs size to conduct the field pilot, and throat diameter ratio is 0.62 at the achieved hydration equilibrium state. With appropriate size, PM800 could smoothly inject into the reservoir, progressively migrate to the deep reservoir, and execute deep plugging. Other physical and injection properties are shown in Table 3.

Theoretical analysis and experimental evaluation show that PM800 has good adaptability to WYQ and excellent plugging performance. The injection concentration is chosen to be 5000 ppm based on the formula of injection concentration and block rate. Additionally, the injection volume is about 29 tons and the injection period is 160 d. We assume the best injection rate is consistent with the original water injection rate of 42 m^3^/d. The injection parameters are shown in Table 4.

### 4.3. Oil Recovery Comparison

**Figure 13:**
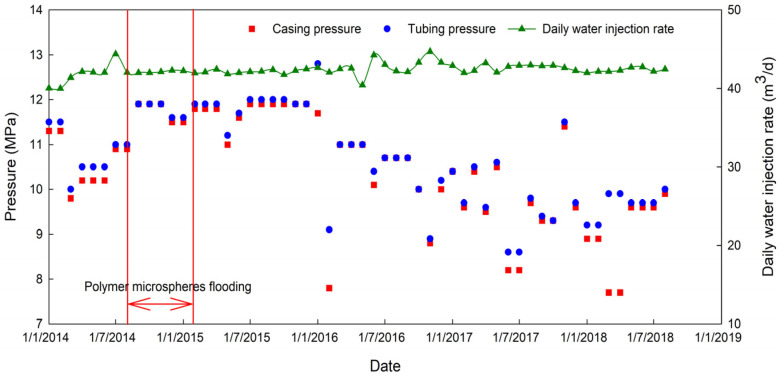
Daily water injection rate, casing pressure and tubing pressure.

The main parameters of development performance analysis include changing the injection pressure trends of the injection wells, tubing and casing, and the water injectivity presented by the daily water injection rate. In order to study the pressure change trend during and after the PM800 injection, the tubing, casing pressure and the actual injection volume are monitored. The monitored data are shown in Figure 13. Figure 13 shows that during the PM800 injection, there was no significant change in the daily injection rate, and it remains the same as an initial injection rate of 42 m^3^/d, indicating that the injection capacity has not decreased.

However, the tubing pressure and casing pressure increase significantly. The increasing pressure indicates that the increasing injection pressure is a crucial indicator parameter for the effectiveness of PMs’ conformance control. The average injection pressure rises to 1.6 MPa, which indicates that the PMs formed an effective plugging between the injection and production wells.

Increased sweep efficiency leads to increased oil production. With limited plugging strength, if the injection water breaks through the plugging area, the flow around injection water is not apparent, and the PMs’ validity period ends. The validity period starts in July 2015 and ends in February 2017. 

**Figure 14:**
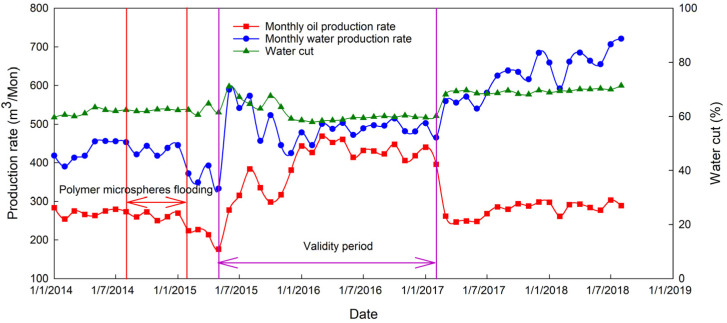
Monthly oil and water production rate and water cut.

In this pilot test of PMs’ conformance control, the total oil production volume during the validity period is 8368.5 t. The oil production rate of the selected well groups is shown in Figure 14. According to the former geology study, the reserves of well control of the selected well group is 68.75 × 10^4^ t, and the actual oil recovery calculated with production data is 1.22%. The oil recovery obtained from the proposed model is 1.37%, the oil displacement efficiency is 0.42, the vertical sweep efficiency is 0.76, and the areal sweep efficiency is 0.043. Comparing the two oil recoveries, it can be found that the theoretical oil recovery value is larger than the oil recovery calculated with field data, and the error is 12.3%, i.e., within the acceptable range.

## 5. Conclusions

An effective theoretical model is established to predict the oil recovery of PMs’ conformance control. The influences of critical factors, including the injection period, injection rate, injection concentration, and PMs’ size, are investigated. Additionally, the model’s validity is verified by comparing it with field application data. The following conclusions can be drawn from this study.

(1) The recovery factor calculation model can quickly and accurately evaluate the oil recovery performance of polymer microspheres’ conformance control. The equivalent mobility ratio was introduced to optimize the vertical sweep efficiency calculation method. A trapezoidal flow tube is proposed to characterize the streamline shape after PMs’ conformance control, and to calculate the areal sweep efficiency.
(2) A series of adaptability analyses on the injection period, injection rate, injection concentration, and PMs’ size are conducted in order to reveal how these functional parameters affect results. The results indicate that long-term injection of small size and low concentration benefits higher oil recovery.
(3) The theoretical oil recovery obtained from the proposed model is 1.37%, and the actual oil recovery of field application is 1.22%, with an error of 0.15%. The recovery factor calculated by the theoretical model agrees better with the field application. This model not only provides a faster and more accessible tool to predict and evaluate the production performance of the PMs’ flooding process, but also could be used to optimize the injection scheme of PMs’ conformance control.

## Figures and Tables

**Table 1:** Parameters and design values for sensitivity analysis.

No.	Factors	Parameter Design Value	Default Value
1	Injection period (d)	50, 70, 90, 120, 150	90
2	Injection rate (m^3^/d)	25, 30, 35, 40, 45	40
3	Injection concentration (ppm)	2000, 3000, 4000, 5000, 6000	5000
4	Polymer microsphere size (µm)	0.1, 0.3, 0.8, 5, 10, 20	0.1

**Table 2:** Support parameters for sensitivity analysis.

No.	Parameters Name	Default Value	No.	Parameter Name	Default Value
1	Well spacing (m)	300	12	Expansion ratio	5
2	*m*	1	13	Irreducible water saturation (f)	0.322
3	*C*	0.0864	14	Split coefficient of injection volume (f)	0.25
4	Inner zone permeability (10^−3^ µm^2^)	30	15	Blocking rate (f)	0.4
5	Outer zone permeability (10^−3^ µm^2^)	25	16	Oil displacement efficiency (f)	0.3
6	Crude oil viscosity (MPa)	1.80	17	Vertical sweep efficiency (f)	0.42
7	Porosity (f)	0.2	18	Permeability variation coefficient (f)	0.4
8	Threshold pressure gradient (MPa/m)	0.01	19	Water viscosity (MPa)	0.46
9	Hole diameter (m)	0.1	20	Equivalent mobility ratio	10
10	Polymer microsphere density (g/cm^3^)	1.2	21	Recovery of polymer microsphere (f)	0.85
11	Water flooding recovery (f)	0.4	22	Production pressure differential (MPa)	25

**Table 3:** Reservoir and fluid properties.

No.	Parameter Name	Default Value	No.	Parameter Name	Default Value
1	Well spacing (m)	360	12	Expansion ratio	2.6
2	*m*	1	13	Irreducible water saturation (f)	0.322
3	*C*	0.0864	14	Split coefficient of injection volume (f)	0.125
4	Inner zone permeability (10^−3^ µm^2^)	131	15	Blocking rate (f)	0.8
5	Outer zone permeability (10^−3^ µm^2^)	18.32	16	Oil displacement efficiency (f)	0.42
6	Crude oil viscosity (MPa)	1.95	17	Vertical sweep efficiency (f)	0.76
7	Porosity (f)	0.12	18	Permeability variation coefficient (f)	0.37
8	Threshold pressure gradient (MPa/m)	0.01	19	Water viscosity (MPa)	0.46
9	Hole diameter (m)	0.1	20	Equivalent mobility ratio	1.8
10	Polymer microsphere density (g/cm^3^)	1.1	21	Recovery of polymer microsphere (f)	0.86
11	Water flooding recovery (f)	0.4	22	Production pressure differential (MPa)	24

**Table 4:** PMs injection scheme of PM800.

Parameter	Value
Size (nm)	800
Concentration (ppm)	5000
Rate (m^3^/d)	42
Injection period (d)	160

## Data Availability

The data presented in this study are available from the corresponding authors upon reasonable request.
